# Supplementary material for: Mechanistic insights into transcription factor cooperativity and its impact on protein-phenotype interactions
Source: Nat Commun. 2020 Jan 8;11:124. doi: 10.1038/s41467-019-13888-7 (PMC6949242; doi:10.1038/s41467-019-13888-7)
Supplement: Supplementary file 2 — Supplementary Information [file 41467_2019_13888_MOESM2_ESM.pdf]

Supplementary Information

Mechanistic insights into transcription factor cooperativity and its impact on protein-phenotype interactions

Ignacio L. Ibarra[1,2], Nele Merret Hollmann[1,2], Bernd Klaus[3], Sandra Augsten[1], Britta Velten[3], Janosch Hennig[1] & Judith B. Zaugg[1,4].

[1]Structural and Computational Biology Unit, European Molecular Biology Laboratory, Heidelberg Germany

[2]Collaboration for joint PhD degree between EMBL and Heidelberg University, Faculty of Biosciences

[3]Genome Biology Unit, European Molecular Biology Laboratory, Heidelberg Germany

[4]Lead contact (e-mail: judith.zaugg@embl.de)

Supplemental Figures

Supplementary Figure 1. Analysis of tiled *k*-mers length and prediction models in SELEX data.

Related to Figure 1 (**a**) Depiction of tiled *k*-mer approach applied to HT-SELEX data analyzed by Yang *et al*[1]. Briefly, reference *k*-mers defined in this work are tiled increasingly, and the correlation between ΔR^2 estimates from *1mer+shape* versus *1mer* models is calculated. (**b**) Spearman correlation between tiled *k*-mers and reference *k*-mers. Red line indicates ρ = 0.5, and threshold for selection of tiled *k*-mers in CAP-SELEX data. (**c**) ΔR^2 values observed for Forkhead+Ets datasets after removing k-mers containing FHL sequence (GACGC)[2] from Forkhead datasets (**Methods**). Asterisks as defined in *Fig 1c*. (**d**) Pairwise comparison between *1mer+shape+2merE2* versus *1mer+shape+2merE2*, *1mer+2mer+3mer* vs. *1mer*, *1mer+shape+3merE2* vs. *1mer+shape+2merE3*, and *1mer+shape+3merE2* versus *1mer+2mer+3mer*. Points are highlighting TF-families per CAP-SELEX dataset as in Figure 1b. (**e**) Comparison between improvements per family group for Forkhead+Ets CAP-SELEX datasets for several models, versus *1mer* models. Asterisks are indicated as in *Fig 1c*.

Supplementary Figure 1

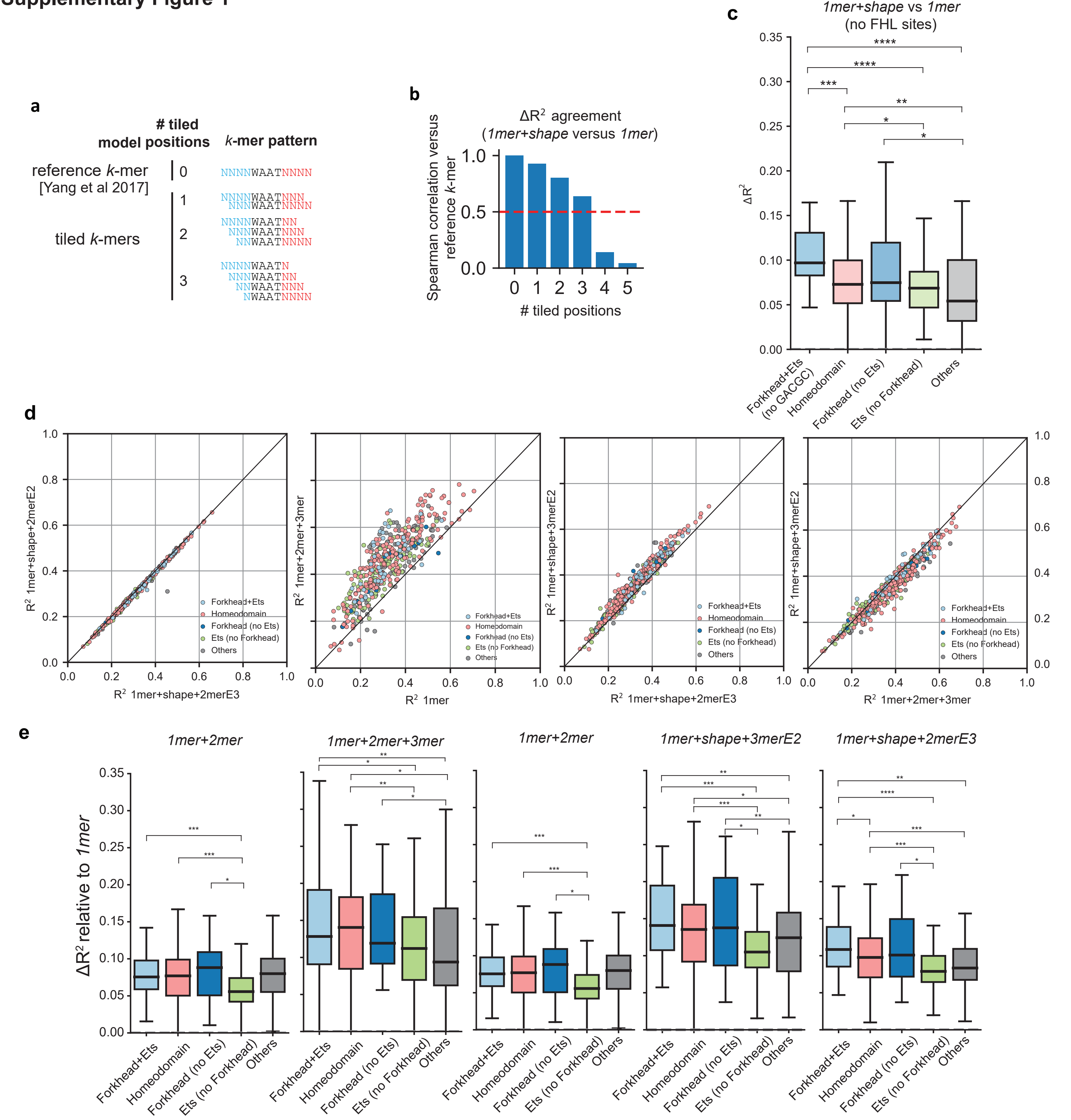

Supplementary Figure 2 Forkhead and Forkhead-Ets structures with Fkd and FHL DNA sequences

Related to Figure 2. (**a**) FOXO1:ETS1:Fkd (green/orange and gray) crystallized complex (PDB ID = 4bl0). DNA nucleotides in red indicate start and end positions of RYAAACA motif (Fkd), and yellow indicates intermediate nucleotides (**b**) structures of FOXO1:Fkd (green/black) and FOXN3:FHL (magenta/gray) complexes (PDB ID = 3co6 and 6cne, respectively)[3,4] (**c**) Superposition of FOXO1:ETS1:Fkd complex with FOXO1:Fkd ((i) +(ii)) and FOXN3:Fkd ((i) +(iii)) with using Forkhead DNA-binding domain as a reference for structural alignment (function `align` in PyMOL). For FOXN3:FHL, red nucleotides in DNA indicate start and end of GACGC site, and yellow indicate intermediate nucleotides (d) Sequences *ω*-none, *ω* and *ω*-high as studied in this work. Nucleotides in red indicate start and ending positions for DNA binding domain binding regions (Fkd for *ω*-none and *ω*, FHL for *ω*-high). The start nucleotide for *ω*-high is shifted by one position with respect to *ω*/*ω*-none (expected no shift), and the Ets binding sites are aligned, suggesting a +1 spacing between Forkhead-Ets DNA-binding domains in ω-high with respect to *ω*-none/*ω*.

Supplementary Figure 2

a

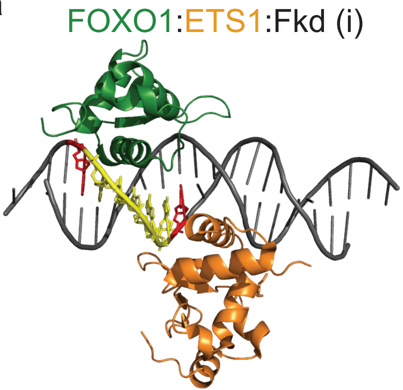

b

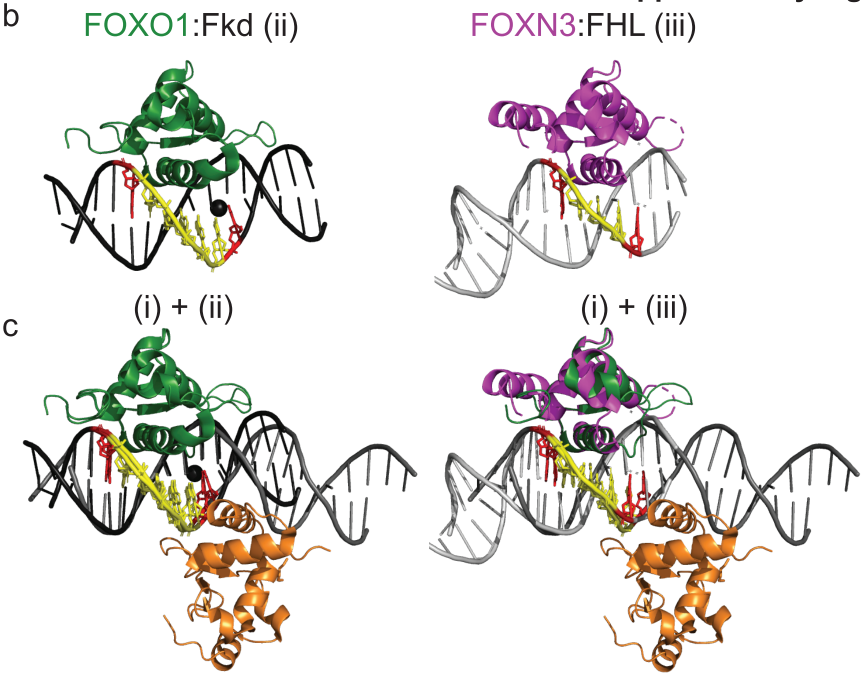

d

ω-none	TGTAAACAGGAAGT
ω	AAACAACAGGAAGT
ω-high	GACGCACCGGAAGT

Supplementary Figure 3. Protein Binding Microarray and SELEX comparison for *k*-mers containing cooperative and non-cooperative Forkhead-Ets sequences.

(**a**) Protein Binding Microarray E-scores distributions for Forkhead family members. Each boxplot shows a group of *8*-mers containing the *7*-mer in Forkhead region (ω-none = GTAAACA; ω = AACAACA; ω-high = ACGCACC). Red line indicates 0.35, threshold that defines high affinity sites. Asterisks indicate $P < 0.001$ (Wilxocon rank sum test. two-sided) (**b**) Average relative affinities from CAP-SELEX data for 10-mers within 13-mers related to ω-none/ω/ω-high. From left to right: other datasets (not including Forkhead, nor Ets), Ets without Forkhead, Forkhead without Ets and Forkhead+Ets datasets. Dots in the Forkhead+Ets group indicate averaged relative affinity for FOXO1:ELK1 using related 10-mers. Asterisks indicate Wilcoxon rank sum test between Forkhead+Ets group and other groups (two-sided), with Benjamini Hochberg correction. (**c**) (*left*) Relative affinity values for 10-mers containing patterns related to ω-none and ω. X-axis indicates increasing HT-SELEX selection rounds. The distribution of relative affinities for *k*-mers containing the high affinity FOXO1 site is inverted at round 3 and 4 with respect to initial rounds. (*right*) Relative affinities for 10-mers obtained from CAP-SELEX data from FOXO1 and TF partners. X-axis indicates experiment ID, including TF1, TF2, selection round and barcode ID. 6 out of 9 comparisons show no differences in relative affinities distributions. Statistical comparisons were done using independent *t*-test, with Benjamini Hochberg correction procedure.

Supplementary Figure 3

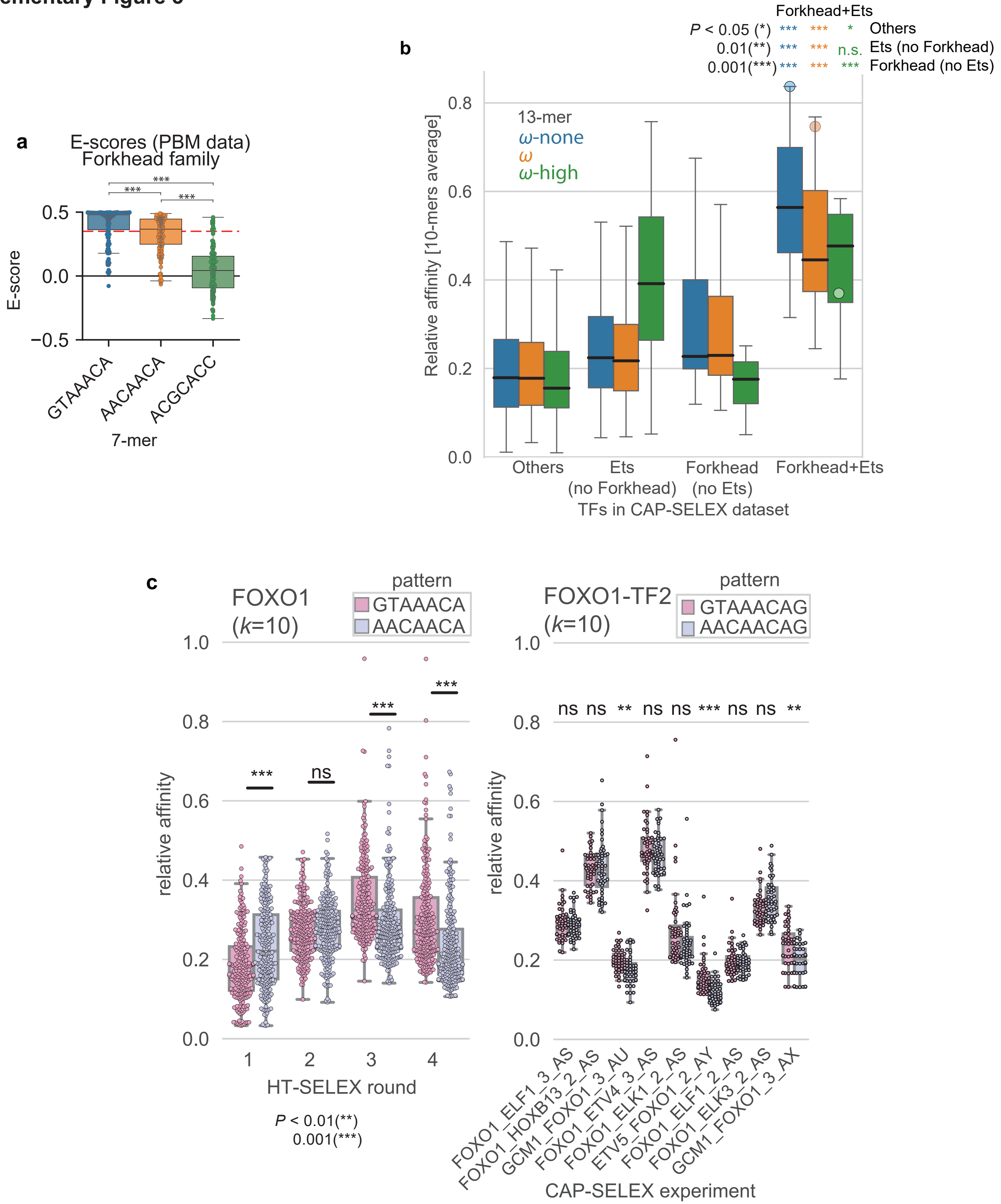

Supplementary Figure 4. FOXO1:ETS1 binding against cooperative and non-cooperative DNA sequences with ITC

(**a**) (*top*) Titration scheme. FOXO1 (green) is titrated from the syringe into a cell with DNA (strands) (*middle*) ITC calorimetric data for FOXO1 binding to ω. (*bottom*) ITC calorimetric data for FOXO1 binding to ω-none. Values in insets indicate thermodynamic parameter estimates from two replicates. (**b**) (*top*) Titration scheme. FOXO1 is titrated from the syringe into a cell with DNA and ETS1 (yellow). (*middle*) ITC calorimetric data for FOXO1 binding to ω and ETS1 mixture. (*bottom*) ITC calorimetric data for FOXO1 binding to ω-none. and ETS1 mixture. Values in insets indicate thermodynamic parameter estimates from two replicates. (**c**) (*top*) Titration scheme. DNA is titrated from the syringe into a cell with ETS1. (*middle*) ITC calorimetric data for ω-high added to ETS1. (*bottom*) ITC calorimetric data for ω added to ETS1. Values in insets indicate thermodynamic parameter estimates from two replicates (**d**) (*left*) Titration scheme. DNA is titrated from the syringe into a cell with buffer. (*middle*) Heat peaks for ω-high. (*right*) Heat peaks for ω.

Supplementary Figure 4

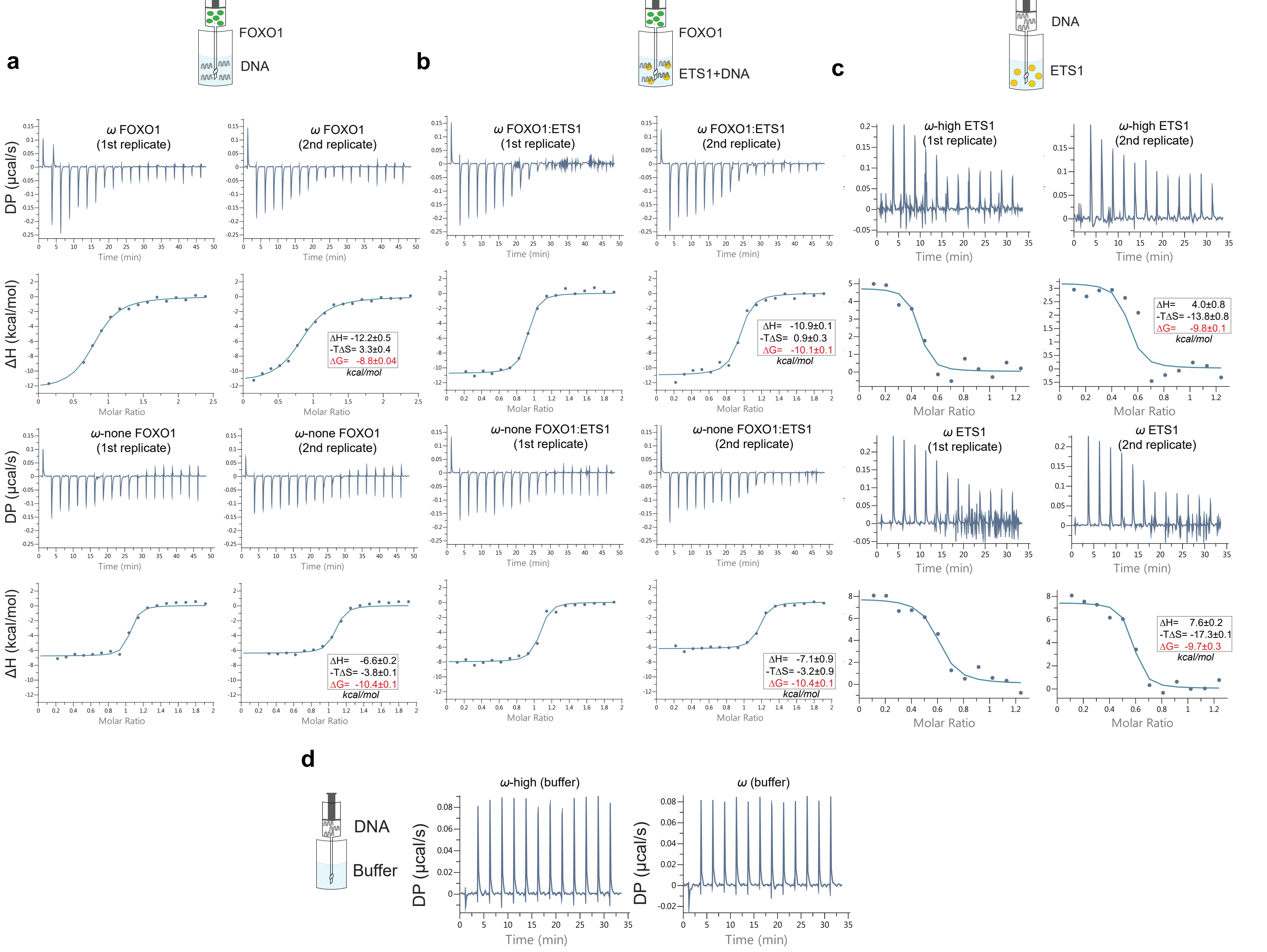

Supplementary Figure 5 ^{1}H-^{15}N HSQC spectra for FOXO1 and *ω*-high without and with ETS1

Related to Figure 2 (**a**) Full spectra for FOXO1 interacting with *ω*-high. Colors indicate DNA to protein concentration ratios (**b**) Selected spectra from insets in (*a*) (**c**) Full spectra for FOXO1:ETS1 interacting with *ω*-none. (**d**) Zoom views of insets in (*c*).

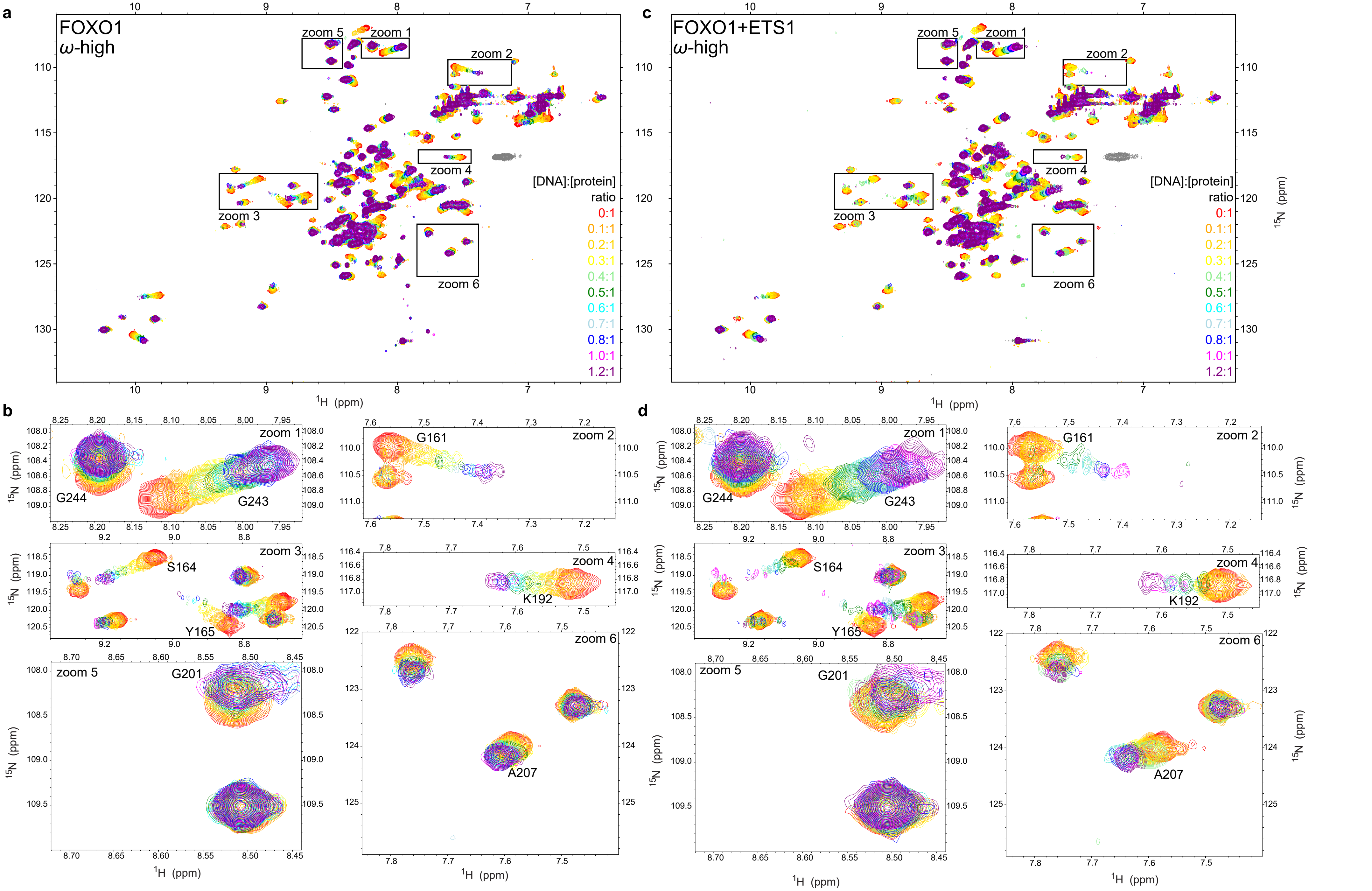

a
FOXO1
ω-high
zoom 1
zoom 2
zoom 3
zoom 4
zoom 5
zoom 6
[DNA]:[protein] ratio
0:1
0.1:1
0.2:1
0.3:1
0.4:1
0.5:1
0.6:1
0.7:1
0.8:1
1.0:1
1.2:1
1H (ppm)
15N (ppm)
b
G244
G243
G161
S164
Y165
K192
G201
A207
c
FOXO1+ETS1
ω-high
d

Supplementary Figure 6 ^{1}H-^{15}N HSQC spectra for FOXO1 and *ω*-none without and with ETS1

Related to Figure 2 (**a**) Full spectra for FOXO1 interacting with *ω*-none. Colors indicate DNA to protein concentration ratios (**b**) Selected spectra from insets in (*a*) (**c**) Full spectra for FOXO1:ETS1 interacting with *ω*-none. (**d**) Zoom views of insets in (*c*).

Supplementary Figure 6

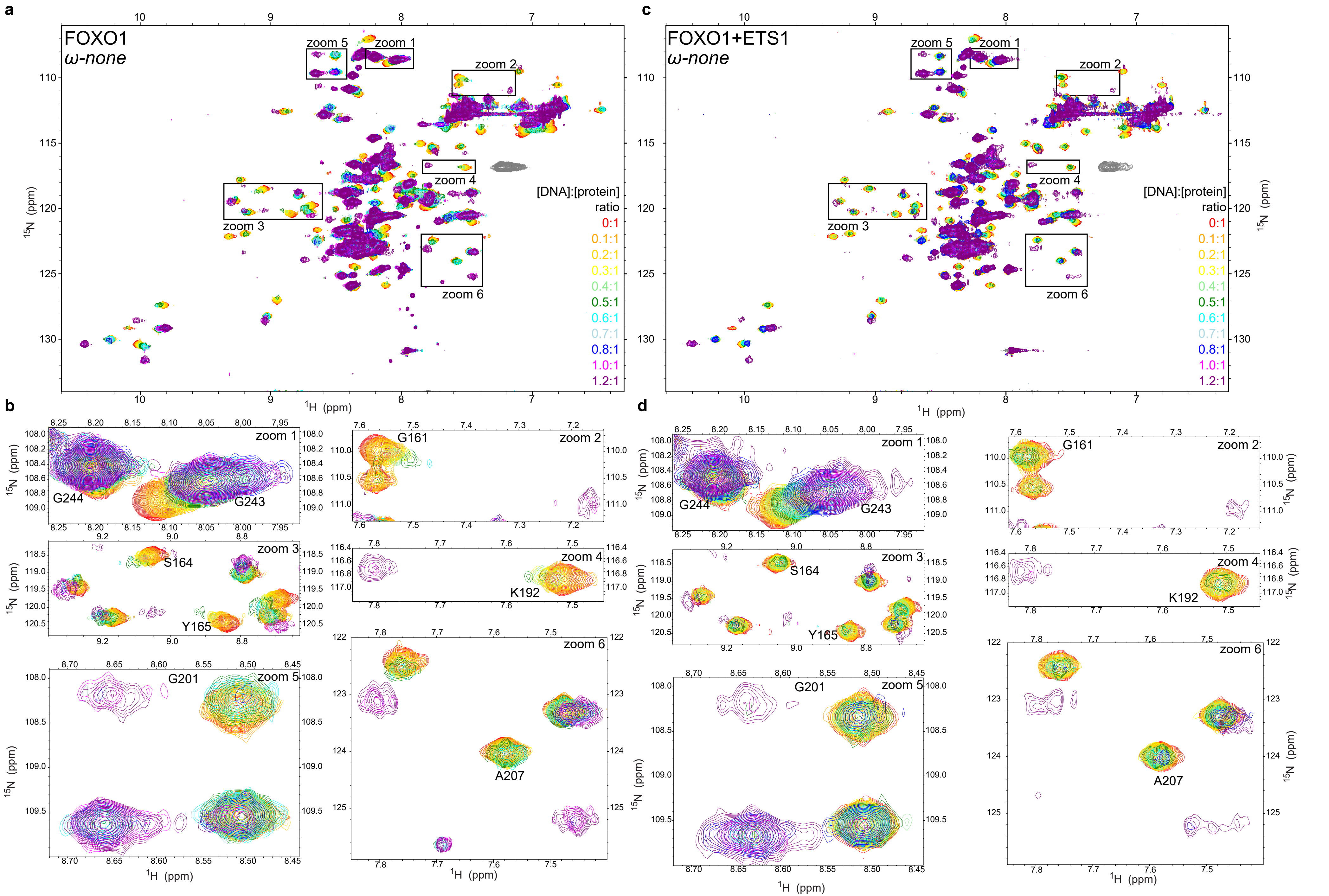

Supplementary Figure 7 DNA-structure features for studied Forkhead-Ets binding sites

Related to Figure 4. Four representative DNA-structure features and one physicochemical feature are highlighted for each of the studied Forkhead-Ets DNA binding sites. Values are shown as heatmap (*top*) and line plots (*bottom*). MGW=Minor Groove Width, PROT=Propeller Twist, HELT=Helical Twist. Black line shape parameters obtained for the DNA molecule within the FOXO1:ETS1:DNA complex (PDB ID = 4lg0), using Curves+[63]. Highlighted region indicates positions where Forkhead+Ets performances changes in CAP-SELEX data are the highest. (*bottom right*) Root-squared absolute deviations (Δshape)[6] for observed differences between positions 1 and 5, for *ω*-none versus *ω* and *ω*-none versus *ω*-high. Z-scores (parentheses) indicate normalized deviation of this value versus differences obtained in 1000 permutations with scrambled versions of ω-none and matched versions of *ω* and *ω*-high in the first 10 positions.

Supplementary Figure 7

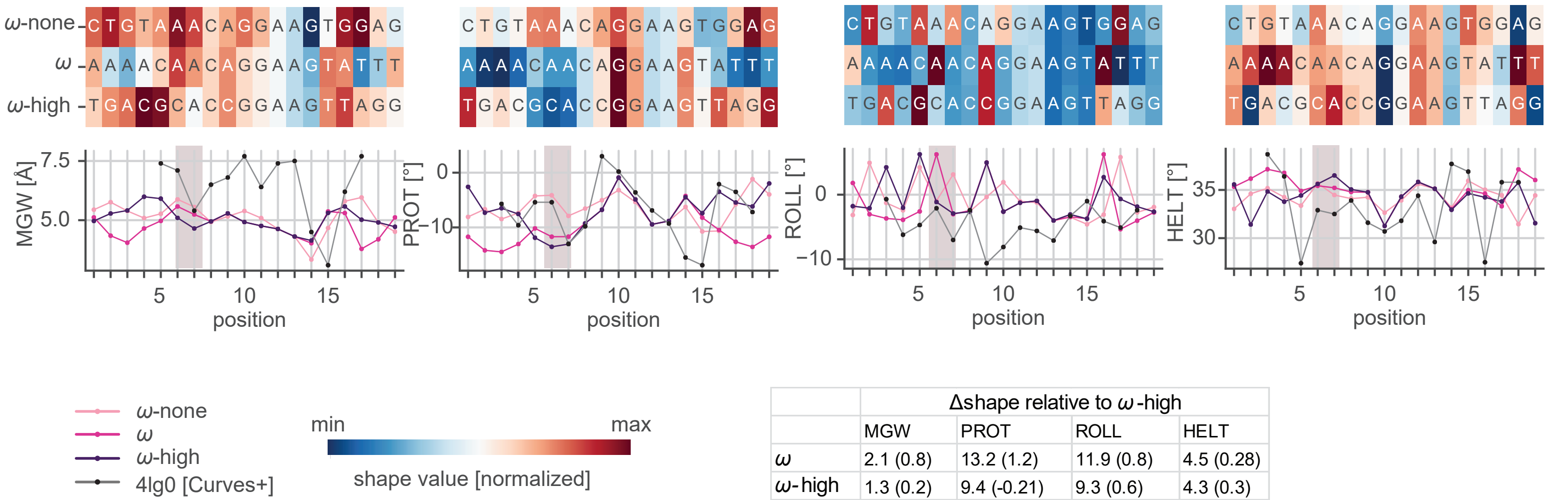

	Δshape relative to ω-high			
	MGW	PROT	ROLL	HELT
ω	2.1 (0.8)	13.2 (1.2)	11.9 (0.8)	4.5 (0.28)
ω-high	1.3 (0.2)	9.4 (-0.21)	9.3 (0.6)	4.3 (0.3)

Supplementary Figure 8. ETS1 mutant R409A binding for Forkhead-Ets cooperative DNA sequence measured with ITC.

(**a**) (*left*) Scheme indicating ITC experiment to measure ETS1 binding to DNA sequences. DNA (strands) is in the syringe and added into a cell with ETS1 mutant (yellow). (*right*) Calorimetric curve for ETS1 mutant R409A binding to DNA (**b**) Comparison of values generated for WT replicates (**Supplementary Figure S4b**), and mutant R409A. Line indicates 95% CI

Supplementary Figure 8

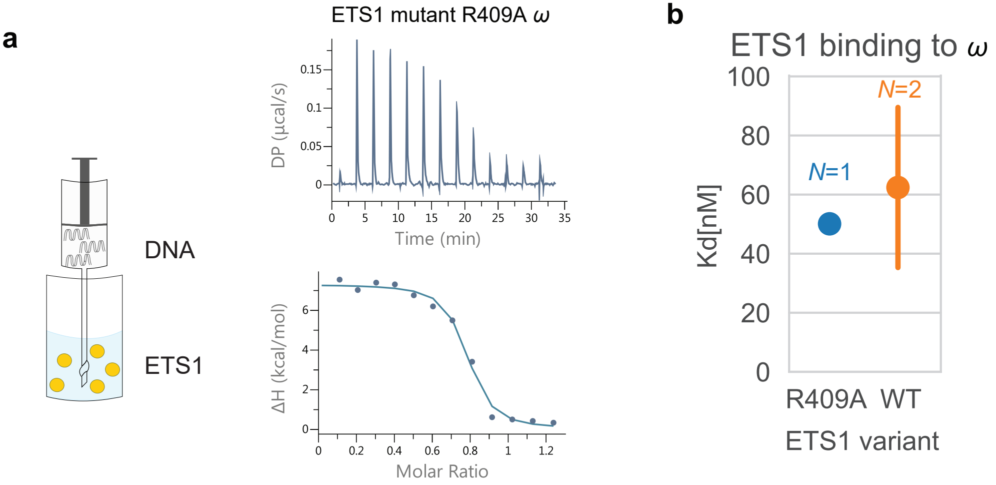

Supplementary Figure 9. Benchmark of improvements per position consistency in HT-SELEX data.

Related to Figure 3 (**a**) tiled *k*-mers defined as shorter versions of the core motifs in HT-SELEX data analyses are used to study agreements observed in positional improvements by addition and removal of DNA-shape features. The estimated positional effect of all *k*-mers is compared with the effect observed with the model that uses the longest *k*-mer, using correlations (**b**) Distribution of correlations for all observed datasets (median Spearman correlation=0.31). (**c**) (*upper*) POU2F2 motif generated from top 100 *k*-mers by relative affinity (reference *k*-mer = NNNNWAATNNNN) (*middle*) Comparison between improvements per position generated using reference *k*-mer (blue line) and tiled *k*-mers (orange line) (Spearman correlation = 0.85) (*bottom*) Heatmap depicting ΔR^2_p values obtained for each tiled *k*-mer. Stronger biases are consistently obtained between positions one and five (**d**) Comparison of L2-coefficients for Minor Groove Width Features in CEBPB binding model obtained from HT-SELEX data, tiling 3 positions from reference *k*-mer. (*top*) CEBPB motif generated as in (*c*). (*middle*) L2-coefficients for tiled *k*-mer aligned to that region of the reference *k*-mer. (bottom) Number of times coefficients are in agreement for the same sign in each position.

Supplementary Figure 9

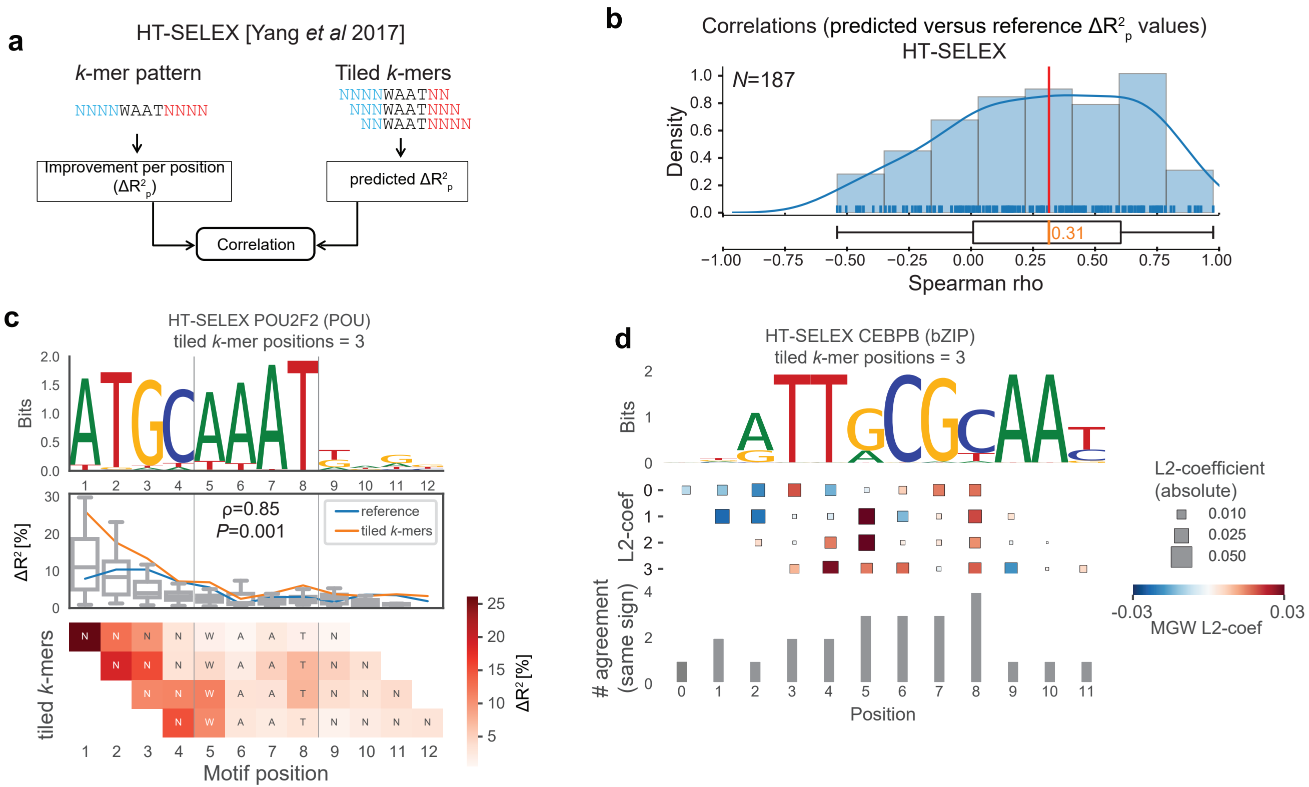

Supplementary Figure 10 Positional improvements in CAP-SELEX data and Forkhead+Ets interactions

(**a**) Analysis of TF families enriched during PAM clustering of shape profiles for increasing number of clusters. Plateau is reached at five (three TFs families and two TFs. Enrichment values in (**Supp Table**) (**b**) *Related to Fig 3b*. (*upper*) Distribution of CAP-SELEX datasets by TF family (*lower*) enrichment values (* = p-value < 0.05. Black box = p-adjusted value < 0.1) (**c**) Absolute changes in R^2 for CAP-SELEX models containing FOXO1, using individual DNA-shape properties. (**d**) *Related to 3c*. (*right*) Comparison between relative R^2 FOXI1 in CAP-SELEX (blue) and values obtained from HT-SELEX data (orange line) (**e**) *Related to 3d*. Reference *k*-mers of FOXI1 datasets aligned, and comparison of positional improvements after alignment (**f**) *Related to 3f*. Dissociation constant measurements for FOXO1 to ω alone (N=2) upon addition of ETS1 WT (N=2) or mutant Y410A (N=1).

Supplementary Figure 10

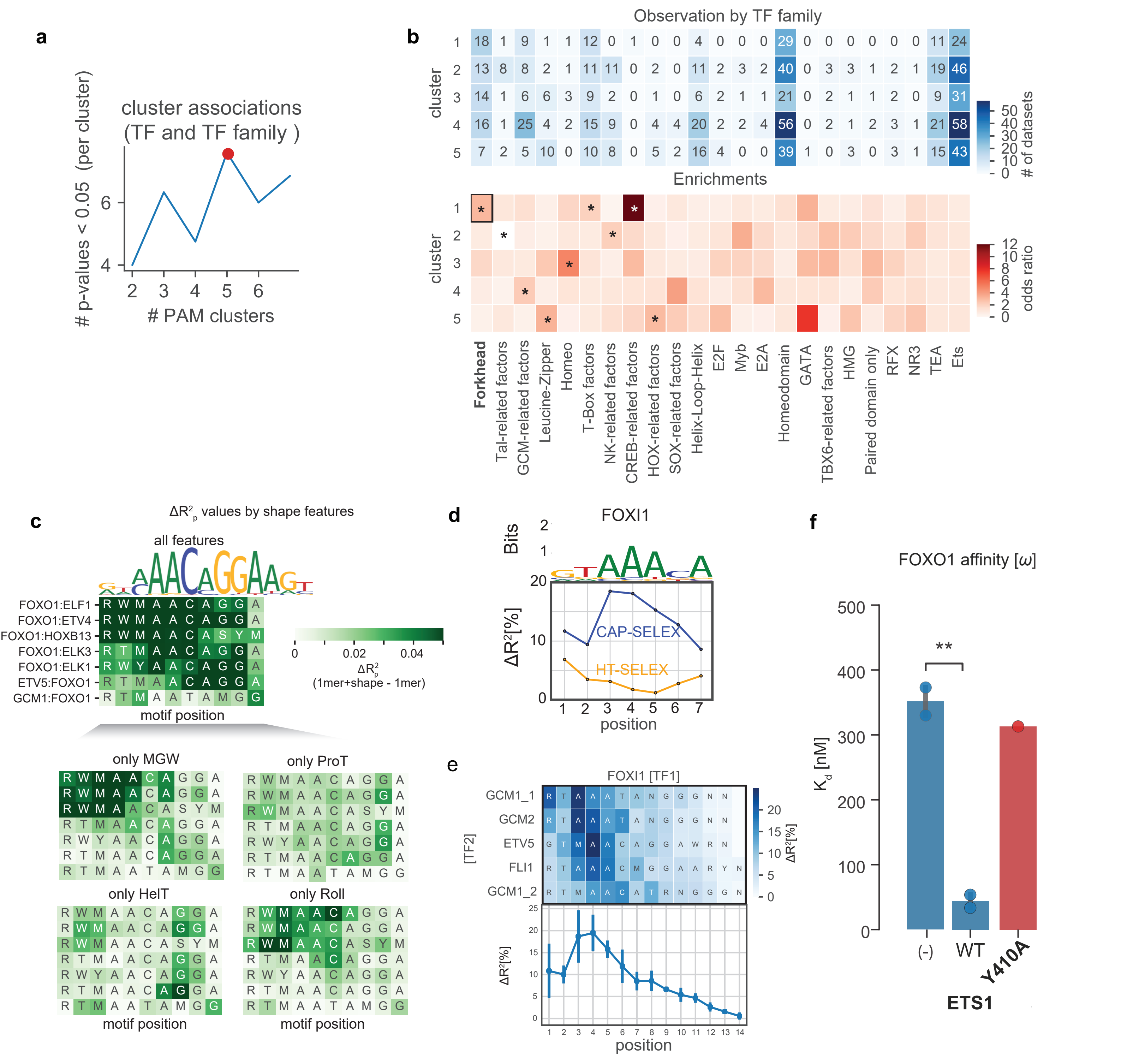

Supplementary Figure 11 TF-TF associations to phenotypes using Ontology Association Probability.

Related to Figure 5 (**a**) *TF1 or TF2* classification benchmark results, using ontology association probabilites to predict those associations with respect to *background* terms. Legend, error bars and *P*-values as defined in *Fig 5c*. (**b**) Five strongest associations by ontology database using the category *TF1 and TF2* to assign TF pairs to ontologies. Red line indicates mean association probabilities for decoy terms (**c**) *Related to Figure 6* (*top*) Ontologies related to differentiation and disease show a strong association to specific TF-TF pairs where at least one TF is known be related to that particular process. (*bottom*) Motifs related to each column are highlighted and grouped by their TF-TF names. When more than one topology exists, the motif with the highest score is highlighted in a black rectangle. References TFs in bold are highlighted[7–9] (**d**) Kaplan-Meier plot of time-to-treatment in CLL patients subset by IGHV and p53 mutation statuses (*N*=88). Data, *P*-values and expression groups for FOXO1 and ETV6 are defined equivalently to *Fig 6b*.

Supplementary Figure 11

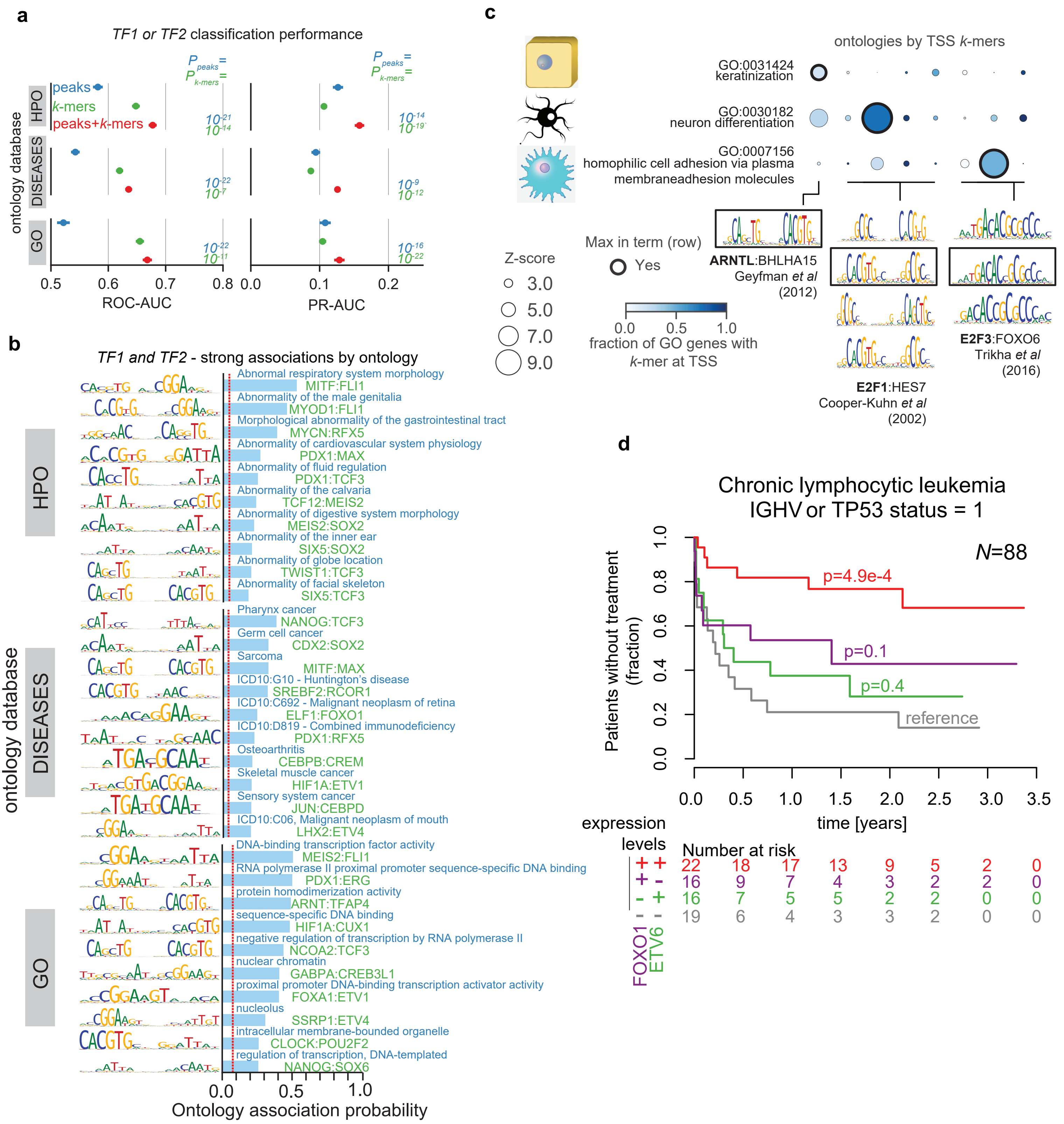

Description of Additional Supplementary Files

Supplemental Data 1. CAP-SELEX datasets used in this study for trim-and-summarize R^2 calculations, and values by model type.

Supplemental Data 2. DNA-shape features for trimers and tetramers, used for flanking positions in *shape* models.

Supplemental Data 3. NMR data and ITC concentration parameters

Supplemental Data 4. TF family and TF enrichments results using five PAM clusters, including HT-SELEX data used for comparison with CAP-SELEX for FOXO1, FOXI1 and FOXO1:ELK3 and sequences used for alignment of ETS members.

Supplemental Data 5. Results for *PWM+shape* models using TF-TF data, correlations between *in vitro* and *in vivo* positional performance changes~~.~~,

Supplemental Data 6. Co-enrichments for *ω*-none and *ω* in Forkhead-Ets ChIP-seq co-occupied peaks.

Supplemental Data 7. Benchmark results for Ontology Association Probabilities and *Z*-scores and strong TF-TF and ontology associations.

Supplementary References

1. Yang, L. *et al.* Transcription factor family-specific DNA shape readout revealed by quantitative specificity models. *Mol. Syst. Biol.* **13**, 1–14 (2017) 10.15252/msb.20167238.
2. Nakagawa, S., Gisselbrecht, S. S., Rogers, J. M., Hartl, D. L. & Bulyk, M. L. DNA-binding specificity changes in the evolution of forkhead transcription factors. *Proc. Natl. Acad. Sci. U. S. A.* **110**, 12349–54 (2013) 10.1073/pnas.1310430110.
3. Rogers, J. M. *et al.* Bispecific Forkhead Transcription Factor FoxN3 Recognizes Two Distinct Motifs with Different DNA Shapes. *Mol. Cell* (2019) doi:10.1016/j.molcel.2019.01.019 10.1016/j.molcel.2019.01.019.
4. Brent, M. M., Anand, R. & Marmorstein, R. Structural Basis for DNA Recognition by FoxO1 and Its Regulation by Posttranslational Modification. *Structure* **16**, 1407–1416 (2008) 10.1016/j.str.2008.06.013.
5. Blanchet, C., Pasi, M., Zakrzewska, K. & Lavery, R. CURVES+ web server for analyzing and visualizing the helical, backbone and groove parameters of nucleic acid structures. *Nucleic Acids Res.* **39**, W68–W73 (2011) 10.1093/nar/gkr316.
6. Wang, X. *et al.* Analysis of Genetic Variation Indicates DNA Shape Involvement in Purifying Selection. *Mol. Biol. Evol.* **35**, 1958–1967 (2018) 10.1093/molbev/msy099.
7. Geyfman, M. *et al.* Brain and muscle Arnt-like protein-1 (BMAL1) controls circadian cell proliferation and susceptibility to UVB-induced DNA damage in the epidermis. *Proc. Natl. Acad. Sci. U. S. A.* **109**, 11758–11763 (2012) 10.1073/pnas.1209592109.
8. Cooper-Kuhn, C. M. *et al.* Impaired adult neurogenesis in mice lacking the transcription factor E2F1. *Mol. Cell. Neurosci.* **21**, 312–323 (2002) 10.1006/mcne.2002.1176.
9. Trikha, P. *et al.* E2f3 in tumor macrophages promotes lung metastasis. *Oncogene* **35**, 3636–3646 (2016) 10.1038/onc.2015.429.
