# Supplementary material for: Mechanistic insights into transcription factor cooperativity and its impact on protein-phenotype interactions
Source: Nat Commun. 2020 Jan 8;11:124. doi: 10.1038/s41467-019-13888-7 (PMC6949242; doi:10.1038/s41467-019-13888-7)
Supplement: Supplementary file 3 — Description of Additional Supplementary Files [file 41467_2019_13888_MOESM3_ESM.pdf]

Description of Additional Supplementary Files

Supplemental Data 1. CAP-SELEX datasets used in this study for trim-and-summarize R^2 calculations, and values by model type.

Supplemental Data 2. DNA-shape features for trimers and tetramers, used for flanking positions in *shape* models.

Supplemental Data 3. NMR data and ITC concentration parameters

Supplemental Data 4. TF family and TF enrichments results using five PAM clusters, including HT-SELEX data used for comparison with CAP-SELEX for FOXO1, FOXI1 and FOXO1:ELK3 and sequences used for alignment of ETS members.

Supplemental Data 5. Results for *PWM+shape* models using TF-TF data, correlations between *in vitro* and *in vivo* positional performance changes.,

Supplemental Data 6. Co-enrichments for *ω*-none and *ω* in Forkhead-Ets ChIP-seq co-occupied peaks.

Supplemental Data 7. Benchmark results for Ontology Association Probabilities and *Z*-scores and strong TF-TF and ontology associations.
